# Non-O1/non-O139 *Vibrio cholerae* bacteraemia in mainland China from 2005 to 2019: clinical, epidemiological and genetic characteristics

**DOI:** 10.1017/S0950268820001545

**Published:** 2020-07-08

**Authors:** Xinyao Li, Yuanyuan Wu, Xiaojun Sun, Jianping Ma, Xiaofeng Li, Cuiping Liu, Hongxiang Xie

**Affiliations:** 1 Department of Cardiology, Zhejiang Hospital, Hangzhou, China; 2 Department of Clinical Laboratory, Zhejiang Provincial People's Hospital, People's Hospital of Hangzhou Medical College, Hangzhou, China; 3 Department of Shungen Conservative and Endodontic Dentistry, Jinan Stomatology Hospital, Jinan, China; 4 Department of Clinical Laboratory, The First Affiliated Hospital of Shandong First Medical University, Jinan, China; 5 Department of General Medicine, The First Affiliated Hospital of Shandong First Medical University, Jinan, China

**Keywords:** antimicrobial resistance, bacteraemia, China, cirrhosis, non-O1/non-O139 *Vibrio cholerae*

## Abstract

In mainland China, the clinical, epidemiological and genetic features of non-O1/non-O139 *Vibrio cholerae* (NOVC) bacteraemia have been scarcely investigated. Herein, we describe a patient with NOVC bacteraemia diagnosed in our hospital and present a retrospective analysis of literature reports of 32 other cases in China, detailing the clinical epidemiology, antibiotic resistance and molecular characteristics of isolates. Most patients were male (84.8%; median age, 53 years) and had predisposing factors, such as cirrhosis, malignant tumours, blood diseases and diabetes. In addition to fever, gastroenteritis was the most frequent presenting symptom. The mortality rate during hospitalisation was 12.1%. NOVC bacteraemia cases were more common in June–August, with the majority in coastal provinces and the Yangtze River basin. Only 42.4% of cases were attributed to consumption of marine (aquatic) products. Tetracycline, third-generation cephalosporins, and fluoroquinolones were the most effective antimicrobial agents, and the highest frequencies of resistance were recorded for ampicillin/sulbactam (37.5%), amoxicillin/clavulanic acid (33.3%), ampicillin (29.2%) and sulfamethoxazole (20%). Multi-drug resistant isolates were not detected. Limited data indicate that *ctxAB* and *tcpA* genes were absent in all NOVC isolates but other putative virulence genes (*hlyA*, *toxR*, *hap* and *rtxA*) were common. Ten multilocus sequence types were identified with marked genetic heterogeneity between different isolates. As clinical manifestations of NOVC bacteraemia may vary widely, and isolates exhibit genetic diversity, clinicians and public health experts should be alerted to the possibility of infection with this pathogen because of the high prevalence of liver disease in China.

## Introduction

*Vibrio cholerae* is a pathogenic Gram-negative bacillus, which is widely distributed in water environments. *Vibrio cholerae* can be classified into more than 200 serotypes according to the differences of its lipopolysaccharide surface O-antigen. Serotypes, O1 and O139 are associated with classic cholera outbreaks of infection worldwide [1], while non-O1/non-O139 *V. cholerae* (NOVC) isolates do not produce the cholera-causing toxin. Although the clinical significance of these strains has been previously ignored, a number of reports have documented their role in human infections [2, 3].

NOVC most often causes sporadic gastroenteritis and less commonly, parenteral invasive infections. The group of organisms has been estimated to cause 1–3.4% of acute diarrhoeal episodes in both developing and developed countries [3]. NOVC bacteraemia remains rare but has been reported sporadically in a few countries. Despite its large population, clinical and epidemiological data pertaining to NOVC bacteraemia cases are scarce from mainland China with little information on the distribution of virulence-associated genes and genetic relationships among isolates. Herein, we present a case of bacteraemia due to a NOVC strain in an elderly male with underlying alcoholic liver cirrhosis, and a retrospective analysis of related reports of 32 other cases in mainland China.

## Case report

The patient (male, 67 years old) was admitted to the hospital because his ‘two upper limbs had been shaking spontaneously for 1 week, with aggravated salivation in the left corner of the mouth for 1 day’ on 2 July 2019. The shaking manifested at rest, disappeared during activity and sleeping, but increased during tension; excess salivation was evident on the day prior to the onset of shaking. He presented with a history of previous hypertension, alcoholic cirrhosis and diabetes mellitus, but had not been exposed to uncooked seafood or contaminated water, or a history of travel to cholera-endemic areas. On admission, his temperature was 37.0 °C; heart rate, 72/min; blood pressure, 148/77 mmHg. Physical examination revealed icteric skin sclera, mild ascites, splenomegaly, tremor in both upper limbs and positive for Romberg's Sign. Laboratory examination results were as follows: plasma ammonia 131 μmol/l, alanine aminotransferase 19 U/l, aspartate aminotransferase 25 U/l, total bilirubin 36.5 μmol/l, direct bilirubin 18.1 μmol/l, total protein 47.5 g/l, albumin 26.3 g/l, prothrombin time 16.90 s, activated partial thromboplastin time 53.00 s, D dimer 2.26 ml/l, fibrinogen 8.48 ml/l, white blood cell count 3.06 × 10^9^/l, haemoglobin 114.0 g/l and platelet count 46 × 10^9^/l. Ultrasonography showed intraperitoneal effusion, liver cirrhosis, splenomegaly and a hepatopathic gallbladder with multiple gallbladder stones. Chest CT showed a nodular high-density image in the right lower lung field and right pleural effusion. After admission, treatment was administered to improve blood circulation, promote brain metabolism, reduce blood ammonia and control blood sugar to alleviate symptoms. Vomiting and diarrhoea had occurred once after eating meat dumpling on the night of 2 July but was not treated as he did not notify a doctor. On the evening of 6 July, he developed a sudden high fever of 39.2 °C, and emergency laboratory tests showed a white blood cell count of 8.62 × 10^9^/l with 85% neutrophils, and an elevated procalcitonin level of 0.145 ng/ml. A blood sample (10 ml) was taken for culture and the patient was treated empirically with cefoperazone/sulbactam for a suspected bacterial infection.

The blood culture tested positive at 8.5 h and after 24 h culture on blood agar grew *β*-haemolytic, oxidase-positive colonies which on subculture on TCBS agar, appeared as large yellow colonies. *Vibrio cholerae* was suspected and identified by the matrix-assisted laser desorption ionisation-time-of-flight analyser (MALDI-TOF, Bruker). NOVC was identified by slide agglutination tests with polyvalent O1 and O139 antisera, and later confirmed by Shandong Provincial Center for Disease Control and Prevention (CDC). Drugs tested as per CLSI 2016 guidelines showed that the isolate was susceptible to trimethoprim/sulfamethoxazole, third- and fourth-generation cephalosporins, fluoroquinolones and carbapenems. Treatment with cefoperazone/sulbactam was initiated and 2 days later, the patient's temperature returned to normal, with gradual alleviation of symptoms. The patient was finally diagnosed with hepatic encephalopathy, bacteraemia, decompensated alcoholic cirrhosis, hypertension, type 2 diabetes, right lung nodules, right pleural effusion, anaemia, thrombocytopaenia, coagulopathy and peritoneal effusion. He was transferred to the local infectious disease hospital for further treatment, and repeat blood cultures proved negative with no recurrence of fever at 1-month follow-up.

## Literature review

### Methods

A literature review was conducted via an electronic search on PubMed, Web of Science, Embase and Ovid by crossing the keywords ‘*V. cholerae* non-O1’ and ‘bacteremia’. The Boolean operator ‘AND’ was used to add the country for our area-specific search strategy after an initial screen of the literature on NOVC bacteraemia; relevant references of the searched papers were also checked. A search of Chinese-language databases (CNKI, VIP, Wanfang Data) from 1995 through 2019 was also undertaken. Two experienced clinicians reviewed the retrieved articles in full, and after elimination of duplicate studies, we retrieved 24 publications on NOVC bacteraemia from mainland China, all cases were the diagnosis of bacteraemia based on blood culture [4–27], and asked the authors for relevant medical records by E-mail. Including our case, a total of 33 cases were identified in mainland China, all of which were sporadic. Most were case reports, and the largest series comprised five patients. A database was established, and information entered included patient demographics, medical history and risk factors such as environmental exposure, clinical presentation, antimicrobial susceptibility and virulence genes of isolates, treatment, and clinical outcome. QGIS software was used for mapping NOVC isolates in Chinese provinces. Further searches were made of the PubMLST database (http://pubmlst.org/vcholerae/) for multilocus sequence typing (MLST) data to determine the degree of genetic heterogeneity among the isolates. A minimum spanning tree based on the allelic difference among seven housekeeping genes by MLST was constructed using Bionumerics (Applied Maths, Ghent, Belgium).

### Results

#### Demographic and clinical parameters of 33 NOVC bacteraemia cases

**Fig. 1.**
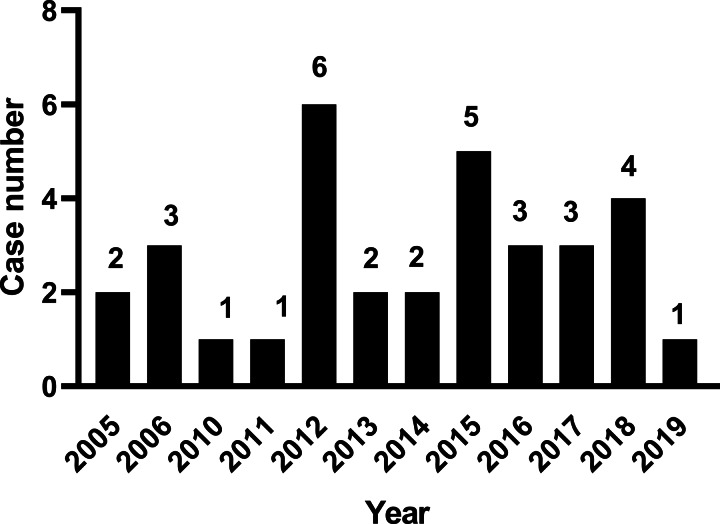
Cases of NOVC bacteraemia reported annually in mainland China.

The first NOVC bacteraemia case was described in 2005, the number of cases peaked in 2012, and 16 cases were reported since 2014 (Fig. 1). The age range was 11 days–89 years (median = 53); 28 males (84.8%) and 5 females (M/F ratio, 5.6:1). Only two cases under 18 years old were reported. The main clinical features are shown in Table 1. The most common risk factors were liver cirrhosis, followed by malignant tumour, haematologic malignancy and diabetes mellitus. Hepatitis B infection was the most common cause of liver cirrhosis (59.1%), followed by chronic alcohol consumption (18.2%). In addition to fever, abdominal pain, diarrhoea, nausea and vomiting were most common. In only 42.4% of cases, the source of infection was associated with the consumption of aquatic products or exposure to contaminated water. Four (12.1%) of the 33 patients died due to NOVC infection during hospitalisation, and two patients abandoned treatment due to serious underlying diseases, giving an estimated overall mortality rate of 18%.

**Table 1.** Demographic and clinical parameters of 32 NOVC bacteraemia in mainland China

Date	Ref. No.	Age/Gender	Underlying diseases/risk factors	Clinical features	Isolate	Stool culture	Treatment	Outcome	Epidemiologic exposure	Region
8/2005	[4]	53/M	Cirrhosis	Fever, chills, lower limbs oedema, bullous cellulitis	Blood	–	–	Death	Skin wound exposed to seawater, seafood ingestion	Zhejiang
8/2005	[4]	51/F	Cirrhosis	Fever, diarrhoea	Blood	–	–	Survival	Exposure to seawater, consumption of raw seafood	Zhejiang
6/2006	[5]	37/M	HBC, alcoholic cirrhosis, diabetes mellitus, gallstone	Fever, nausea, vomiting, diarrhoea	Blood	Negative	Gatifloxacin, ceftriaxone	Survival	Iatrogenic infection	Beijing
7/2006	[5]	37/M	HBC, primary liver cancer, diabetes mellitus	Fever	Blood	Negative	Gatifloxacin	Survival	–	Beijing
8/2006	[5]	57/M	HBC, primary liver cancer, diabetes mellitus	Fever, diarrhoea	Blood	Negative	Gatifloxacin, levofloxacin	Survival	Iatrogenic infection	Beijing
8/2010	[6]	47/M	Cirrhosis	Fever, abdominal pain and distension	Blood	Negative	Ciprofloxacin	Survival	–	Sichuan
6/2011	[7]	55/M	HBC	Fever, abdominal pain	Blood	–	–	–	–	Hainan
8/2012	[9]	6/M	Acute lymphatic leukaemia	Weakness, abdominal pain, diarrhoea	Blood	Negative	Meropenem, amikacin	Survival	–	Shanghai
5/2012	[10, 13]	56/M	Cirrhosis	Fever, abdominal distension, ascites	Blood	Negative	Ampicillin	Survival	Pickles infection	Hubei
7/2012	[11]	49/M	HBC, alcohol abuse	Oliguria, ascites	Blood, ascites	Negative	Cefotaxime, levofloxacin	Survival	–	Guangxi
7/2012	[11]	53/M	HBC, alcohol abuse	Fever, abdominal distension, diarrhoea	Blood	Negative	Cefotaxime, levofloxacin	Survival	–	Guangxi
6/2013	[8, 12]	69/M	Alcoholic cirrhosis, hypersplenism, coronary heart disease, atrial fibrillation, hypertension	Chills, fever, abdominal pain, ascites, diarrhoea, jaundice, septic shock	Blood	Negative	Ceftriaxone, levofloxacin	Survival	Seafood ingestion, pickles	Zhejiang
2014	[14]	64/F	Cholangitis, gallbladder stones; chronic cholecystitis, biliary infection	Fever, chills, jaundice	Blood	Negative	–	Survival	–	Fujian
8/2015	[16]	50/F	Myelodysplastic syndrome, pneumonia	Fever, chills, bullous cellulitis	Blood	–	Ciprofloxacin, levofloxacin, imipenem,	Abandon therapy	Chicken ingestion	Hebei
6/2015	[19]	37/M	HBC	Fever, chills, abdominal pain, jaundice, ascites	Blood. Ascites culture negative	Negative	Ceftazidime, levofloxacin	Survival	Consumption of raw Seafood	Jiangsu
9/2012	[15]	70/M	Non-Hodgkin's lymphoma, hypoproteinaemia	Fever, abdominal distension, diarrhoea	Blood	–	Cefoperazone/sulbactam, meropenem, piperacillin/tazobactam	Death	Seafood ingestion	Zhejiang
8/2013	[15]	64/M	Gastric cancer; cirrhosis, oesophageal varices	Fever, chills, diarrhoea	Blood. Ascites culture negative	Negative	Piperacillin/tazobactam	Survival	Seafood ingestion	Zhejiang
7/2015	[15]	43/M	HBC, hepatic encephalopathy, oesophageal varices,	Fever, chills, abdominal pain,	Blood	–	Piperacillin/tazobactam, levofloxacin	Survival	Seafood ingestion	Zhejiang
7/2015	[15]	55/M	Alcoholic cirrhosis, oesophageal varices	Fever, chills, diarrhoea,	Blood. Ascites culture negative	Negative	Ceftazidime	Survival	Seafood ingestion	Zhejiang
8/2015	[15]	89/M	Liver dysfunction; common bile duct stones; urinary tract infection	Fever, chills, vomiting, dysuria	Blood	–	Piperacillin/tazobactam	Survival	Seafood ingestion	Zhejiang
4/2016	[17]	37/M	HBC	Fever, chills, abdominal distension	Blood	Negative	Ceftazidime, levofloxacin	Survival	–	Jiangsu
8/2016	[21]	66/M	Lung cancer, cirrhosis	Fever	Blood	–	–	Death	Saltwater fish ingestion	Shandong
9/2016	[18]	59/M	HBC	Fever, diarrhoea	Blood	Negative	Cefoperazone/sulbactam, amikacin	Survival	Crayfish, pickles ingestion	Jiangsu
6/2017	[20]	37/M	HBC	Fever, diarrhoea, abdominal pain, nausea, vomiting, jaundice, ascites	Blood	Negative	Piperacillin/sulbactam	Survival	–	Jiangsu
7/2017	[20]	65/M	Cholangiocarcinoma	Fever, chills	Blood	–	Cefepime	Survival	–	Jiangsu
10/2017	[20]	28/M	HBC	Fever, abdominal distension, ascites, jaundice, shock	Blood	–	Levofloxacin	Abandon therapy	–	Jiangsu
6/2018	[24]	55/M	Cirrhosis	Fever, nausea, vomiting, jaundice	Blood	Negative	Ceftazidime, levofloxacin	Survival	–	Anhui
2018	[22]	35/M	Liver dysfunction	Abdominal pain, vomiting, diarrhoea, shock	Blood	–	–	Death	Bean jelly	Guizhou
9/2018	[23]	47/M	Non-Hodgkin's lymphoma, hepatitis B	Fever, chills, vomiting, diarrhoea	Blood	Negative	Meropenem	Survival	Chicken ingestion	Sichuan
2019	[27]	46/F	Aplastic anaemia, connective tissue disease, hepatitis B	Fever, nausea, vomiting, diarrhoea	Blood	Negative	Imipenem cilastatin	Survival	Seafood ingestion	Shandong
9/2012	[25]	70/M	Diabetic nephropathy	Fever, chills	Blood	Negative	Meropenem	Survival	Hairtail ingestion	Beijing
7/2014	[26]	0/F	Infant	Meningitis	Blood	–	Flucloxacillin, meropenem, sulbecillin, metronidazole	Survival		Shandong

M, male; F, female; HBC, hepatitis B-caused liver cirrhosis; –, not available or missing information

#### Antimicrobial resistance of isolates

Antimicrobial susceptibility testing data were not uniform due to the variation in numbers of isolates and the range of agents tested. However, resistant isolates were identified for trimethoprim/sulfamethoxazole (6 of 30 cases), ampicillin (7/24), ampicillin/sulbactam (3/8), amoxicillin/clavulanic acid (3/9), cefazolin (1/6), cefepime (1/18), ceftazidime (1/24), imipenem (3/28), nalidixic acid (1/2) and polymyxin B (3/3). Multi-drug-resistance was not detected. Isolates fully susceptible to other antimicrobials were as follows: gentamicin (26 tested), amikacin (28), levofloxacin (25), ciprofloxacin (23), piperacillin/tazobactam (18), piperacillin (20), meropenem (16), cefotaxime (16), tetracycline (16), ceftriaxone (14), cefoperazone/sulbactam (9), aztreonam (13), chloramphenicol (9), tobramycin (10), cefoxitin (6), furantoin (6), minocycline (4), cefuroxime (4), gatifloxacin (4), streptomycin (3), doxycycline (3), norfloxacin (3) and ertapenem (3).

#### Spatial and temporal distributions of NOVC isolates

**Fig. 2.**
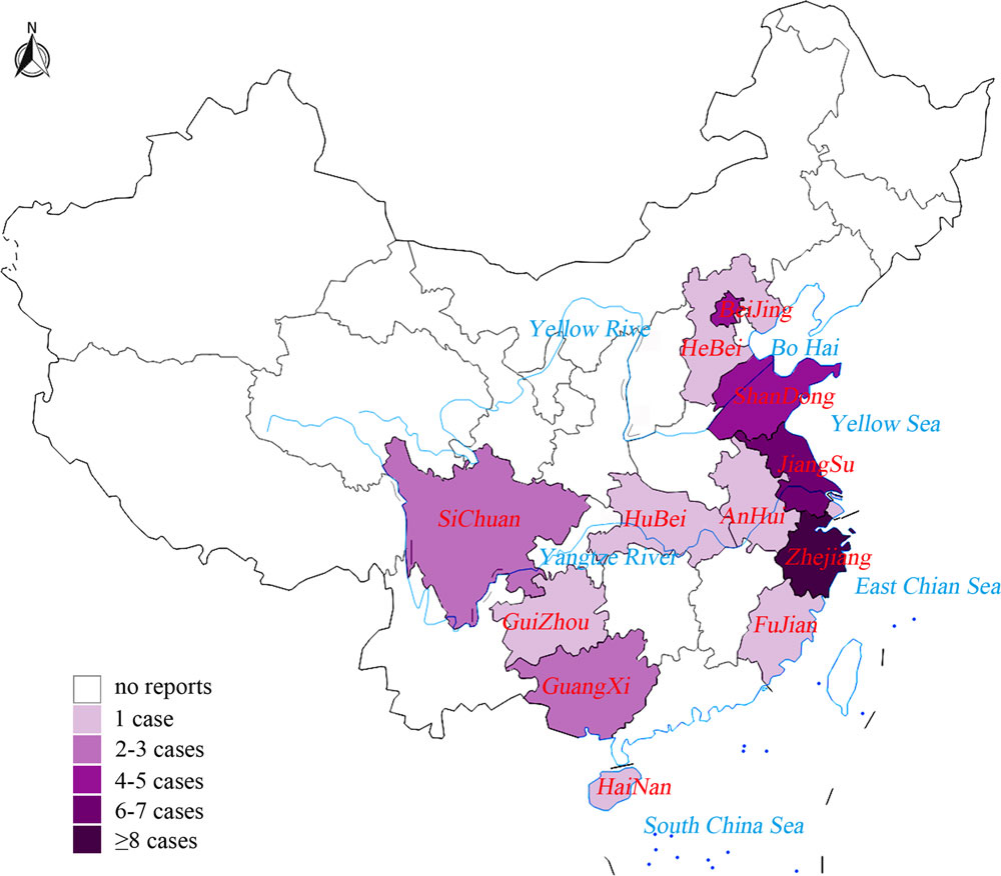
The distribution of NOVC bacteraemia cases between 2005 and 2019 in mainland China according to published literature.

NOVC bacteraemia cases were reported from 13 provinces, the majority being coastal provinces, and the Yangtze River basin (Fig. 2). Cases were reported from April to October, and was most common (23 cases, 70%) in June to August. Four cases were reported in September, and single cases in April, May and October; the isolation month for the remaining three cases was not reported.

#### Distribution of virulence factors genes

Data on virulence genes were available for 15 isolates (Table 2). All were negative for cholera toxin genes. Eleven isolates were tested and positive for the *rtx* virulence gene (encoding repeat toxin subunit A or C, respectively), and seven were tested and positive for *hap* (encoding haemagglutinin protease). All, but one, of 10 isolates carried *hlyA* (encoding El Tor-like haemolysin), and 6/7 harboured *toxR gene* (encoding CT transcriptional activator). None of the tested isolates was positive for type III secretion system (T3SS) genes. Miscellaneous isolates proved positive for various other virulence genes (Table 2).

**Table 2.** Details of NOVC strains reported in the literature and PubMLST database

Isolate	year	region	*ctxAB*	*tcpA*	other virulence factors	ST
1407	2014	Shandong	–	–	*rtxC+ hlyA+ hap+ toxR− dth+ ompW+ ompU+ chxA+ PrtV+ T3SS−*	188
BSVC01	2013	NA	–	–	*rtxA+ T3SS− stn−*	206
120	2014	Shandong	–	–	*rtxA− T3SS− stn−*	207
147	2014	Shandong	–	–	*rtxA− T3SS− stn−*	208
RA03	2016	Zhejiang	–	–	*rtxA+ hlyA+ hap+ toxR+ nanH+ T6SS+ T3SS− zot− ace−*	267
RA01	2016	Zhejiang	–	–	*rtxA+ hlyA+ hap+ toxR+ nanH+ T6SS+ T3SS− zot− ace−*	268
RA02	2016	Zhejiang	–	–	*rtxA+ hlyA+ hap+ toxR+ nanH− T6SS+ T3SS− zot− ace−*	269
RA04	2016	Zhejiang	–	–	*rtxA+ hlyA+ hap+ toxR+nanH+ T6SS+ T3SS− zot− ace−*	270
RA05	2016	Zhejiang	–	–	*rtxA+ hlyA+ hap+ toxR+ nanH+ T6SS− T3SS− zot− ace−*	271
KY1143	2014	Beijing	–	–	*rtxA+ hlyA+ mshA+ ST+ IS1004+ T3SS−*	107
NA	2018	Guizhou	–	–	*rtxC+ hlyA+ Hap+ toxR+ lolB+ ompW− PrtV+ ompU*	NA
NA	2012	Hubei	–	–	*rtxC+ hlyA− toxR+*	NA
NA	2016	Fujian	–	NA	*rtxC+ hlyA+ ompW+*	NA
NA	2016	Jiangsu	–	NA	NA	NA
NA	2012	Shanghai	–	NA	NA	NA

NA, not available or missing information.

#### Molecular typing

**Fig. 3.**
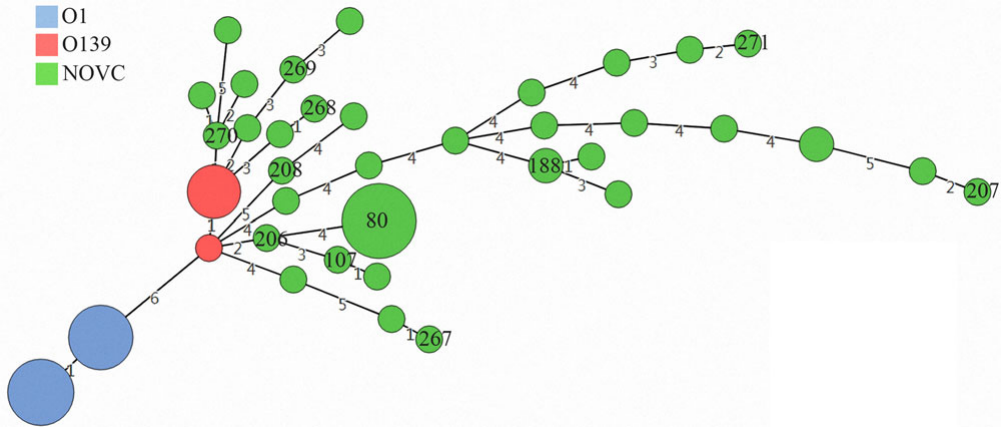
Minimum spanning tree analysis of NOVC isolates based on sequence type (ST). Ths STs are designated by the number in the circle; the size of the circle corresponds to the total number of each ST. The digits on the lines between the two circles represent the number of allelic differences.

MLST data were reported for 10 isolates, all of which were unique (Table 2). None of them belonged to ST80 which was the dominant ST reported from China of NOVC recovered from diarrhoeal stools [28]. The 10 isolates showed high diversity and were distinct from their toxigenic O1 and O139 counterparts (Fig. 3). Most STs differed from each other by three or more loci, with no evidence of clustering according to year or geographic location.

## Discussion

Following the identification of a case of NOVC bacteraemia in our hospital (Shandong province), we conducted a retrospective case series analysis of reports of 32 cases, in addition to our case, in mainland China from 2005 to 2019. Where reported, data were extracted on risk factors, epidemiology, clinical presentations and mortality as well as genetic characterisation of reported causative isolates. A review of the related literature in mainland China showed that the incidence of NOVC bacteraemia is higher in summer and autumn, and predominantly affects middle-aged males, and rarely children. Our case also occurred during this season in a 67-year-old man with mild diarrhoea and vomiting, as reported for other cases. It is not known why men are more susceptible to NOVC infection than women, but a similar trend has been noted in other *Vibrio* infections [29]. Recently, it has been suggested that the pro-inflammatory effect of oestradiol may reduce the incidence of some bacterial infections and associated complications in women, while the susceptibility of males may be associated with testosterone-mediated immunosuppression [30]. Additionally, behavioural and social factors such as differences in consumption levels of seafood and alcohol, and aquatic exposure may also explain this gender-related variation.

In the 350 cases of NOVC bacteraemia reviewed by Deshayes *et al*. [3], most cases were identified in patients with underlying diseases, especially liver disease/cirrhosis and immunosuppressed conditions. In mainland China, the most common risk factor was liver cirrhosis, followed by malignant tumour, haematologic malignancy and diabetes mellitus, which is consistent with Deshayes' report. Susceptibility to NOVC bacteraemia in patients with liver cirrhosis may be related to anatomical and physiological changes including high intestinal mucosal permeability due to inflammation and oedema, by-pass of the hepatic reticuloendothelial system by portal hypertension, complement deficiencies, impaired phagocytosis, and alterations in iron metabolism and/or inefficient chemotaxis [31]. It has also been suggested that liver disease and haematological malignancy are often accompanied by low platelet count or abnormal coagulation function, which might facilitate the passage of the bacteria into the systemic circulation, leading to bacteraemia [16]. Recent epidemiological data showed that the trend of liver cirrhosis between gender and age in China was similar to that observed in NOVC cases, with a disproportionate rate of liver cirrhosis in both male and elderly groups [32, 33]. This feature may help to explain the observed gender differences.

The clinical manifestations of NOVC bacteraemia in our series proved to be varied. Fever, abdominal pain and diarrhoea were the most common presentations but three were blood culture-negative and had jaundice and ascites, both of which are indicative of liver cirrhosis, rather than the NOVC infection itself. Diarrhoea was usually watery with no mucus and blood. Deshayes *et al.* reported that a minority (12%) of NOVC bacteraemia cases had bloody (12%) or mucous stools (8%), and almost 5% had abscesses, including hepatic, prostatic, cerebral and peritoneal abscesses [3]. Additionally, pyomyositis, pneumonia, cellulitis, necrotizing fasciitis, endophthalmitis, and meningitis and other rare clinical conditions have been noted by others [34–38]. Our patient had an atypical clinical manifestation of NOVC bacteraemia, except for abnormal liver function tests which were shown by ultrasonography to be due to liver cirrhosis. He had only mild digestive symptoms and the diagnosis mainly depended on blood culture. Stool cultures were performed in 18 cases in the literature review series, all of which were negative for NOVC, despite them having positive blood cultures. It is possible that the stool culture was tested after the start of antimicrobial treatment, leading to the negative result. Indeed, the isolation of NOVC has been reported from various body sites including the respiratory tract, bile, uterus, urine, cerebrospinal fluid, among others [39].

The epidemiology of NOVC bacteraemia has yet to be clarified. Cases might be related to exposure to the aquatic environment or ingestion of aquatic products as the presence of pathogenic vibrios in aquaculture in mainland China has been documented at rates of 0.8–25.96%, with an even higher frequency in circulation and catering links [40–42]. However, the majority (57.6%) of the mainland cases reviewed here did not report a history of consumption of aquatic products or exposure to contaminated water, which suggests that other routes of infection existed. In fact, NOVC have been isolated from wild and domestic animals and in asymptomatic human carriers [2, 43]. Ma *et al.* [16] and Li *et al.* [23] both reported cases of NOVC bacteraemia in patients with malignant blood diseases, and suggested that the most likely source of infection was chicken ingested prior to disease onset. Notably, reports of iatrogenic infections linked to NOVC bacteraemia in mainland China underline the importance of timely detection of the organism and isolation of patients [5]. Our patient had neither recently been exposed to contaminated water nor eaten raw seafood, and hence the source of contamination remains unknown. However, he had consumed a meal of meat dumplings on the day prior to the onset of fever, and in the absence of other information to the contrary, we surmised that ingestion of contaminated food was the most likely source of infection.

The exact mechanism by which NOVC invade the bloodstream remains unclear. MLST analysis showed that isolates from cases had high genotypic diversity and were distinct from the dominant NOVC clone in China (ST80), generally associated with sporadic infections [28]. All strains tested were negative for the virulence-encoding regions of toxigenic *V. cholerae*, such as *ctxAB* or *tcpA*, but possessed several genes encoding putative accessory virulence factors, including *hlyA*, *hap*, *toxR*, *rtxA* and *T6SS*, which might play a role in the disease process. Although *OmpW* gene is highly conserved in *V. cholerae* and can be used for the identification of this microorganism, it is worth noting that the *OmpW* gene might be negative in some NOVC strains. Accordingly, we speculated that these factors might contribute to bloodstream invasion in immunosuppressed conditions, due to production of haemolysin, or cytotoxin (*rtxA* encoded), and their ability to induce cell vacuolation [26]. More recently, it was suggested that naturally occurring IgG recognizing *V. cholerae* outer membrane protein U (OmpU) mediates a serum-killing effect in a complement C1q-dependent manner. Differences in OmpU protein level among different biotypes of *V. cholerae* was considered a reasonable cause for their observed differences in serum resistance, and hence the ability to cause bacteraemia [44]. Additionally, the presence of the cholera toxin genes *ctxA* and *tcpA* in NOVC isolates has been reported in mainland China, which might be related to horizontal transfer of these virulence genes [45]. In our patient case, the blood culture isolate showed *β*-haemolytic colonies, strongly suggesting the production of haemolysin. Additionally, the patient had a poor immune function and coagulation dysfunction, making it easier for the bacteria to breach the patient's immune barrier and enter the bloodstream.

Because NOVC bacteraemia is rare, in the absence of large-scale clinical trials, there are no guidelines for the treatment of such cases. It has been suggested that third-generation cephalosporins or fluoroquinolones are the most suitable agents for these patients but the duration of treatment is also controversial (range 3–75 days; median 14 days). This duration should be adjusted according to the patient's background, clinical manifestation and disease severity (such as meningitis and abscess) [3]. The mortality rate is considered to exceed 25%, and has been significantly associated with respiratory, circulatory or neurological failure [3, 37]. Unlike these patients, our case was an elderly male with comorbidities predisposing him to NOVC bacteraemia. However, his symptoms improved within 2 days after receiving treatment with cefoperazone/sulbactam as a single agent therapy.

Antimicrobial susceptibility testing showed our patient's isolate was susceptible to several commonly recommended antibiotics. We noted that there was significant heterogeneity in the choice, dosage and duration of antimicrobial agents in the published cases from mainland China. Existing data suggest that the administration of tetracycline, cephalosporins and fluoroquinolones remains the best choice. The resistance rates of sulfamethoxazole and ampicillin were unacceptably high, and imipenem also showed less than optimal activity. However, an important factor impeding our reviewed data from being extrapolated to clinical practice was that many authors reported only agents active in their cases, and so, wider documentation of antimicrobial resistance data is required to allow a critical assessment of resistance rates. As most NOVC infections originate from water environments, the influence of intensive use of antibiotics in agriculture and animal husbandry, and the emergence of multidrug-resistant clones, is a cause for concern. Antimicrobial susceptibility testing of naturally occurring and clinical patient isolates therefore remains critical to ensure optimal choice of agent and therapy [46, 47].

In conclusion, NOVC bacteraemia in a minority of cases can prove fatal. As sea surface temperatures continue to rise globally, there is a risk of increased proliferation of pathogenic vibrios in aquatic environments which pose a potential global threat. We should therefore maintain a high level of awareness of these infections especially in patients with a history of liver disease because of the high prevalence of hepatitis B infection in China.

## Data Availability

The datasets generated during and/or analysed during the current study are available from the corresponding author on reasonable request.
